# Fast, robust, and accurate monocular peer-to-peer tracking for surgical navigation

**DOI:** 10.1007/s11548-019-02111-z

**Published:** 2020-01-16

**Authors:** Simon Strzeletz, Simon Hazubski, José-Luis Moctezuma, Harald Hoppe

**Affiliations:** 1 Department of Electrical Engineering, Medical Engineering and Computer Science, Offenburg University, Badstraße 24, 77652 Offenburg, Germany; 2 Stryker Leibinger GmbH & Co. KG, Bötzinger Str. 39–41, 79111 Freiburg im Breisgau, Germany

**Keywords:** Peer-to-peer navigation, Monocular tracking, Pose estimation, Marker assignment

## Abstract

***Purpose*:**

This work presents a new monocular peer-to-peer tracking concept overcoming the distinction between tracking tools and tracked tools for optical navigation systems. A marker model concept based on marker triplets combined with a fast and robust algorithm for assigning image feature points to the corresponding markers of the tracker is introduced. Also included is a new and fast algorithm for pose estimation.

***Methods*:**

A peer-to-peer tracker consists of seven markers, which can be tracked by other peers, and one camera which is used to track the position and orientation of other peers. The special marker layout enables a fast and robust algorithm for assigning image feature points to the correct markers. The iterative pose estimation algorithm is based on point-to-line matching with Lagrange–Newton optimization and does not rely on initial guesses. Uniformly distributed quaternions in 4D (the vertices of a hexacosichora) are used as starting points and always provide the global minimum.

***Results*:**

Experiments have shown that the marker assignment algorithm robustly assigns image feature points to the correct markers even under challenging conditions. The pose estimation algorithm works fast, robustly and always finds the correct pose of the trackers. Image processing, marker assignment, and pose estimation for two trackers are handled in less than 18 ms on an Intel i7-6700 desktop computer at 3.4 GHz.

***Conclusion*:**

The new peer-to-peer tracking concept is a valuable approach to a decentralized navigation system that offers more freedom in the operating room while providing accurate, fast, and robust results.

## Introduction

Optical navigation systems normally consist of one tracking unit and several tracked tools. While the tracking unit usually uses line scan cameras (e.g., Stryker FP 6000) or plane image sensors (e.g., NDI Polaris), the markers are either active [light-emitting diodes (LEDs)] or passive (retroreflective spheres, black-white targets). If at least three markers can be triangulated, the pose of the tracked tool is calculated using point-to-point matching. This concept is “centralized,” and the tracking unit needs an unobstructed view to all tracked objects and therefore has to be placed at a significant distance away from the trackers. This can be problematic in an operating room, where the situs is surrounded by OR personnel. Furthermore, this centralized concept lacks redundancy: The transformation between two tracked tools depends on the unobstructed view to both of them.

In [15], we proposed the novel concept of peer-to-peer trackers which are tracking units and tracked tools at the same time. The tracking of other peers is realized using one camera in conjunction with a novel pose estimation algorithm. In this work, the underlying tracker layout concept is presented which is based on marker triplets together with a fast and robust marker assignment algorithm which assigns the markers found in the camera image to the correct markers of the tracked tools. Furthermore, a new iterative pose estimation algorithm not relying on initial guesses or previously tracked poses is introduced. It is shown that tracking with the proposed concept and the novel algorithms is fast, accurate, and robust even in the presence of accidental marker detections like reflections or unwanted light sources. The correct assignment of markers is of central importance since testing all possible combinations would take too much time.

In contrast to point-to-point matching with at least three point correspondences [1], monocular pose estimation needs at least four point-to-line correspondences (see, e.g., Oberkampf et al. [12]). While Oberkampf presents an iterative pose estimation algorithm for large distances between camera and object, this work focuses on small distances and high accuracy. Furthermore, Oberkampf assigns quality measures to each step of the iteration in order to get the best possible solution. Here, if more than one possible constellation for the same tracker type is found, the smallest residual error of an appropriate objective function is used to find the correct pose of the particular tracker type.

Tjaden et al. [17] use a tracker with seven non-planar LED markers in cross-shape for the marker assignment algorithm. The image features are correctly assigned by analyzing the two lines of the LED cross in the 2D image. Especially in case of highly distorting optics (e.g., fisheye lenses), this requires an image distortion correction. Moreover, Tjaden only introduces one tracker layout and uses a *k*-means approach to distinguish clearly separated clusters of features in the image. The presented algorithms are able to distinguish four different tracker types even if they strongly overlap.

Dornaika and Garcia [6] describe two pose estimation algorithms for weak perspective and paraperspective projection which both assume that the camera exhibits a unique projection center where all viewing rays pass through. The pose estimation algorithm described in “Pose estimation algorithm” section overcomes this restriction and works perfectly for arbitrary constellations. As stated in [12, 14, 19], most pose estimation algorithms show difficulties using planar targets that lead to pose ambiguities and can only be solved with time-consuming calculations. Solutions often lack the ability to truly support feature point assignment under occlusion or with many accidental feature points [17]. This work is robust against such influences.

The presented work was developed for surgical navigation purposes, but is definitely not restricted to this application area. In many different disciplines, methods are needed to determine the location and orientation of objects. These methods have to be fast, cheap, and still accurate enough for the specific fields, e.g., satellite navigation or the calculation of the relative transformation between various unmanned aerial vehicles such as quadrocopters [11]. In recent years, augmented reality applications relying on cheap solutions with fast and accurate transformations between real and virtual objects are on the rise [10].

Teixeira et al. [16] present a solution in which pose estimation with LEDs is used to determine the position and orientation of a flying quadrocopter. They also state that in the field of areal robotics, a cheap solution is needed for pose estimation. Their achieved accuracies lack the requirements for surgical navigation. Faessler et al. [8] use a monocular setup and an iterative algorithm that uses the last detected pose as a starting value for the pose estimation of the next frame. This can result in subsequent errors if the last calculation was incorrect or too old.

**Fig. 1:**
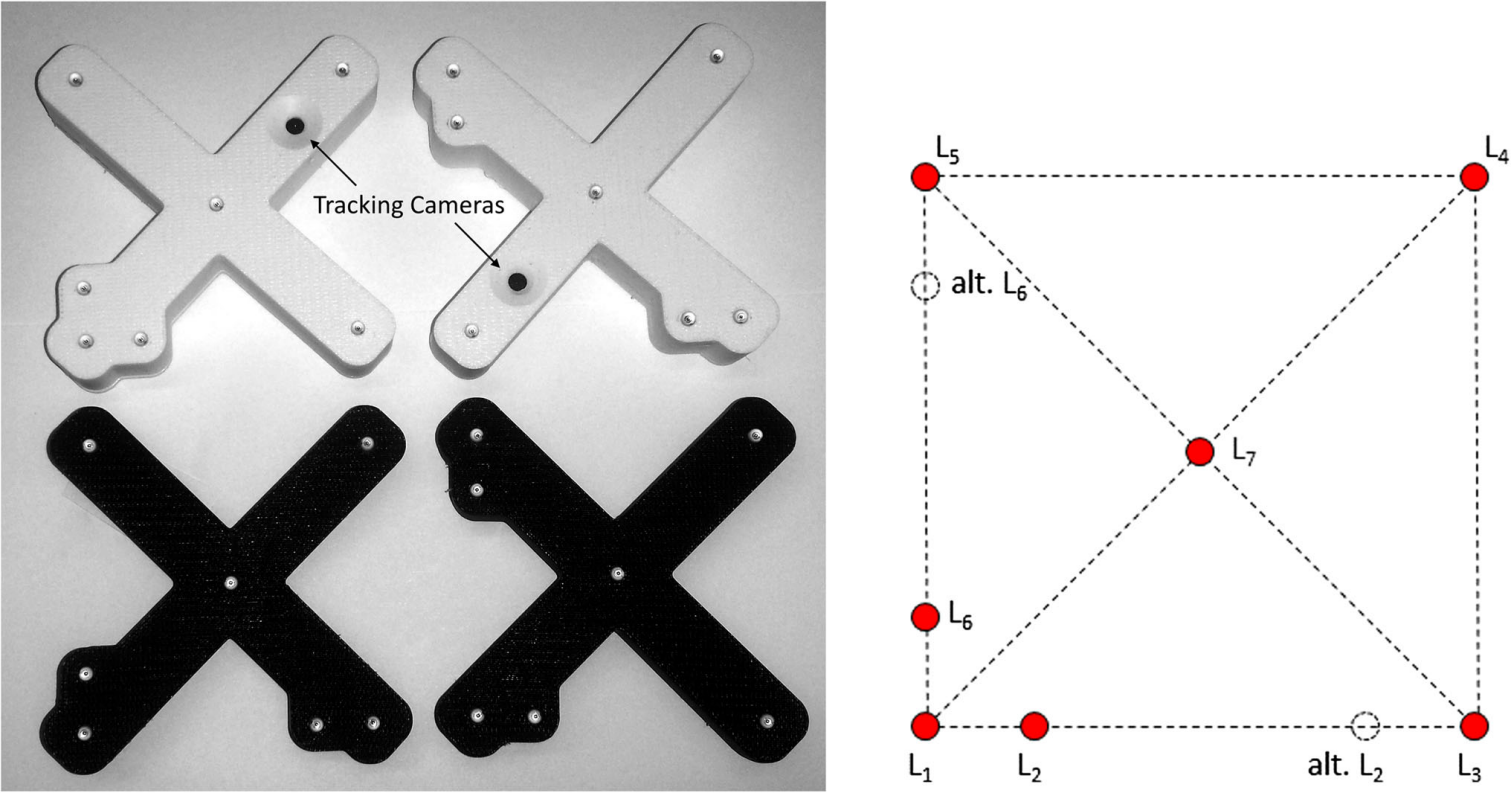
The four possible peer-to-peer trackers with seven markers (LEDs) and one camera (left) and the underlying design concept (right)

The main focus of this work is a fast and robust marker assignment algorithm together with the underlying marker layout concept as well as a novel pose estimation algorithm which works without initial guesses.

## Material and methods

Figure 1 shows the actual design of the proposed peer-to-peer trackers with integrated cameras for pose estimation. The seven markers (LEDs) are arranged in a square configuration with four corners, one middle marker, and two markers ($L_2$ and $L_6$) tagging one of the two outer markers of the respective side triplet. Depending on the position of these tagging markers, four different types of trackers can be realized (see Fig. 1). All in all, each tracker type includes four collinear LED triplets: two side triplets with an asymmetric and two middle triplets with a symmetric LED layout. The cameras are calibrated with the pixel-wise and model-free calibration method introduced in [9]. In [15], it was shown that pose estimation with one camera is accurate enough for surgical navigation purposes.

### Marker assignment algorithm

**Fig. 2:**
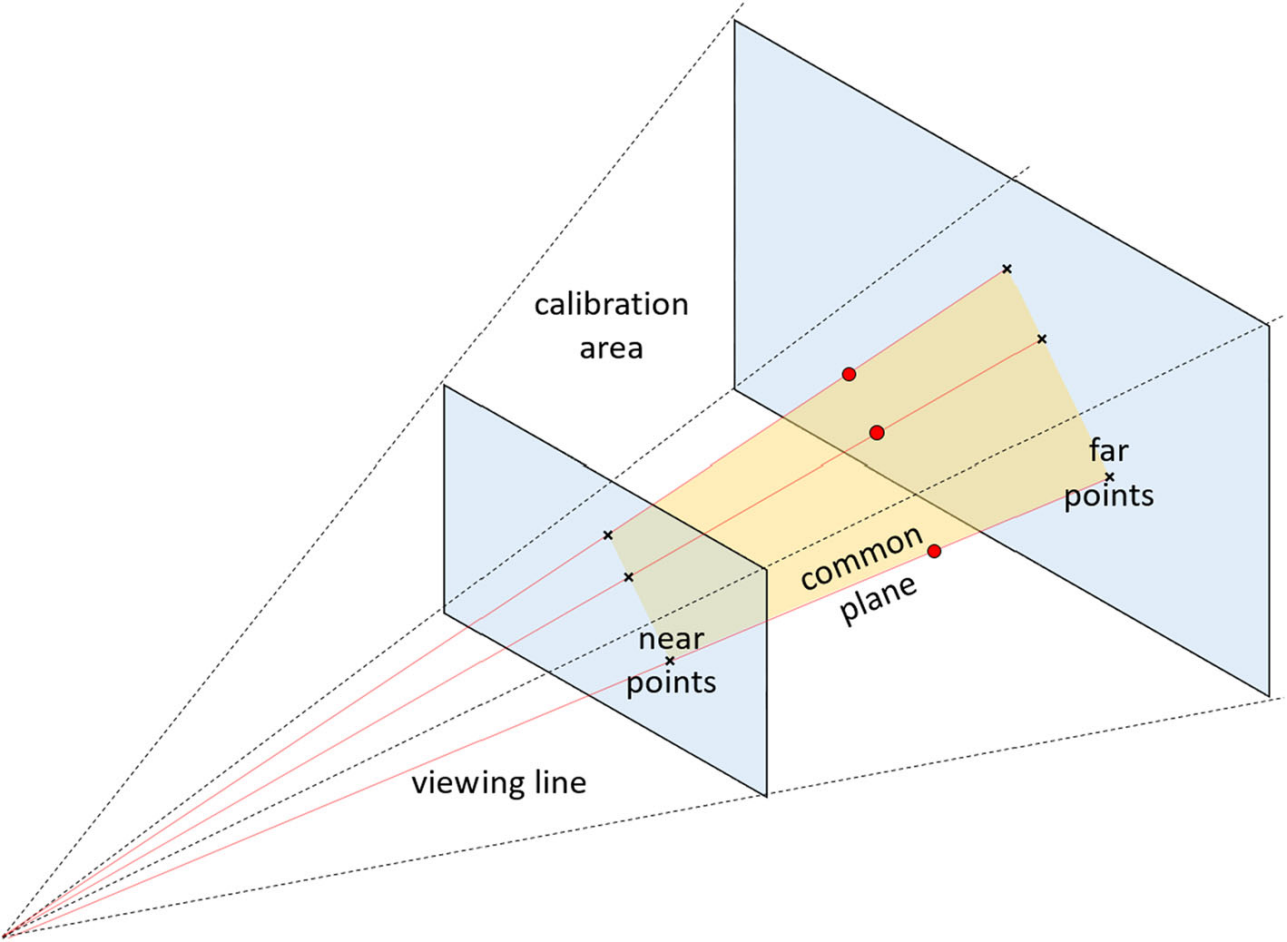
The three near and three far points of a marker triplet lie on a single plane

As stated in “Introduction” section, the fast and correct mapping of image feature points to the markers is crucial for pose estimation. These points are found using a weighted center blob detection which results in a set of $N$ 2D pixel coordinates. Each blob center defines a straight line containing all world points that are projected on the specific pixel coordinates. These straight lines can be defined by two points marking the beginning (near point) and the end (far point) of the calibration area (see Fig. 2).

#### Marker triplets

Given that three markers are collinear, their corresponding viewing lines, and therefore their near and far points, are coplanar (see Fig. 2). All $$\left( {\begin{array}{c}N\\ 3\end{array}}\right) $$ possible combinations of three blobs are investigated by calculating the regression plane through their six near and far points. If the sum of squared distances (SSD) of the points to the regression plane is below a defined threshold $t_\mathrm{SSD}$, the triplet is stored. This results in a set of possible triplet candidates that are processed further on.

#### Marker tagging

**Fig. 3:**
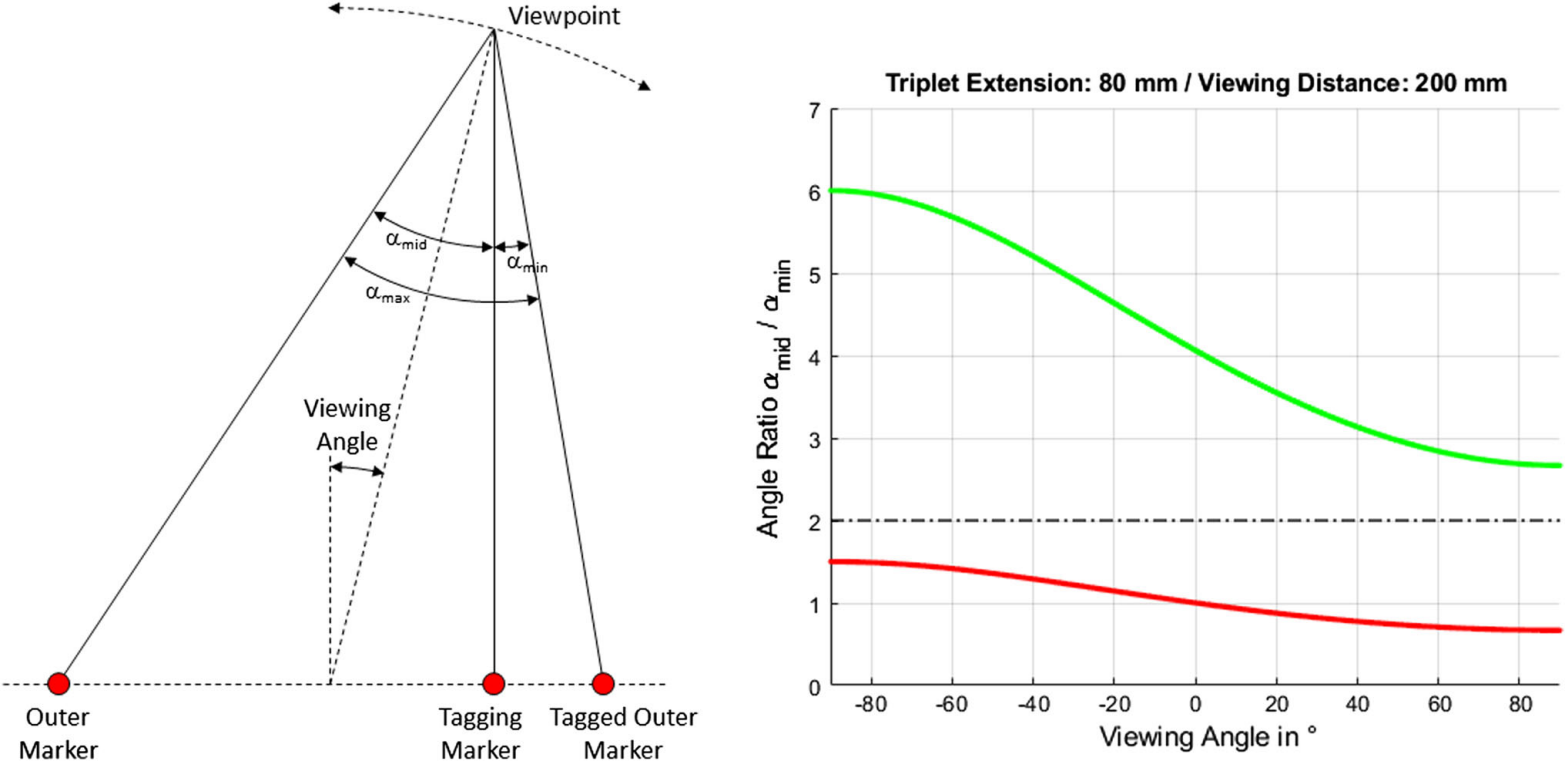
Marker triplet with viewing angles $\alpha _\mathrm{min}$, $\alpha _\mathrm{mid}$, and $\alpha _\mathrm{max}$ (left) and angle ratio $f = \frac{\alpha _\mathrm{mid}}{\alpha _\mathrm{min}}$ for symmetric (red) and asymmetric (green) triplets (right)

The middle marker of a triplet can be used to distinguish between triplets with symmetric and asymmetric layout. In the latter case, the triplet’s middle marker is placed at 20% resp. 80% of the distance between the two outer ones. The differentiation is realized by calculating and sorting the three angles $(\alpha _\mathrm{min}, \alpha _\mathrm{mid}, \alpha _\mathrm{max})$ between the straight lines corresponding to one triplet. The two lines corresponding to the largest angle $\alpha _\mathrm{max}$ correspond to the two outer markers, and the tagged marker also contributes to $\alpha _\mathrm{min}$. See Fig. 3 (left) for further details.

If the triplet is observed from a distance significantly larger than the extension of the triplet, the ratio $f = \frac{\alpha _\mathrm{mid}}{\alpha _\mathrm{min}}$ is 1.0 for symmetric triplets and 4.0 for the chosen percentage of 20% resp. 80%. But the smaller this distance gets, the more this ratio differs from these values. Figure 3 (right) shows $f$ for a triplet extension of 80 mm and a viewing distance of 200 mm for all possible viewing angles. It ranges from 2.7 to 6.0 for asymmetric (green line) and from 0.7 to 1.5 for symmetric triplets (red line). Therefore, $f$ can be used to distinguish the two kinds of triplets: $f<2.0$ for symmetric, $f>2.0$ for asymmetric triplets. Triplets with $f>6.0$ are rejected (the markers align coincidentally).

All in all, this part of the algorithm results in a number of $M$ symmetric middle triplets and $S$ asymmetric side triplets.

#### Combining triplets

Now, all $$\left( {\begin{array}{c}M\\ 2\end{array}}\right) $$ combinations of two middle triplets are checked for a shared middle marker. If a pair was found, the blob IDs are stored as follows:

- $p_\mathrm{id} \ldots$ outer marker of triplet 1 with smaller blob ID
- $q_\mathrm{id} \ldots$ outer marker of triplet 2 with smaller blob ID
- $r_\mathrm{id} \ldots$ outer marker of triplet 1 with larger blob ID
- $s_\mathrm{id} \ldots$ outer marker of triplet 2 with larger blob ID
- $m_\mathrm{id} \ldots$ blob ID of shared middle marker

$p_\mathrm{id}$, $q_\mathrm{id}$, $r_\mathrm{id}$, and $s_\mathrm{id}$ (in this order) define the blob IDs of the corners of the square in clockwise or counterclockwise order. Now, the first side triplet is searched which connects $p_\mathrm{id}$ and $q_\mathrm{id}$, $q_\mathrm{id}$ and $r_\mathrm{id}$, $r_\mathrm{id}$ and $s_\mathrm{id}$, or $s_\mathrm{id}$ and $p_\mathrm{id}$. If found, a second side triplet is searched which shares one corner with the first side triplet. Let $u_\mathrm{id}$ and $v_\mathrm{id}$ be the outer marker blob IDs of this second side triplet. If the first triplet connects, e.g., $p_\mathrm{id}$ and $q_\mathrm{id}$, there are two possible cases for the second side triplet:

1. ($u_\mathrm{id} = p_\mathrm{id}$ and $v_\mathrm{id} = s_\mathrm{id}$) or ($v_\mathrm{id} = p_\mathrm{id}$ and $u_\mathrm{id} = s_\mathrm{id}$) (the second triplet connects $s_\mathrm{id}$ and $p_\mathrm{id}$). Then, corner $p_\mathrm{id}$ is the shared marker and the involved blob IDs are stored in the following order: $p_\mathrm{id}$/tagging marker ID of side triplet 1/$q_\mathrm{id}$/$r_\mathrm{id}$/$s_\mathrm{id}$/tagging marker ID of side triplet 2/$m_\mathrm{id}$
2. ($u_\mathrm{id} = q_\mathrm{id}$ and $v_\mathrm{id} = r_\mathrm{id}$) or ($v_\mathrm{id} = q_\mathrm{id}$ and $u_\mathrm{id} = r_\mathrm{id}$) (the second triplet connects $q_\mathrm{id}$ and $r_\mathrm{id}$). Then, corner $q_\mathrm{id}$ is the shared marker and the involved blob IDs are stored in the following order: $q_\mathrm{id}$/tagging marker ID of side triplet 2/$r_\mathrm{id}$/$s_\mathrm{id}$/$p_\mathrm{id}$/tagging marker ID of side triplet 1/$m_\mathrm{id}$

**Fig. 4:**
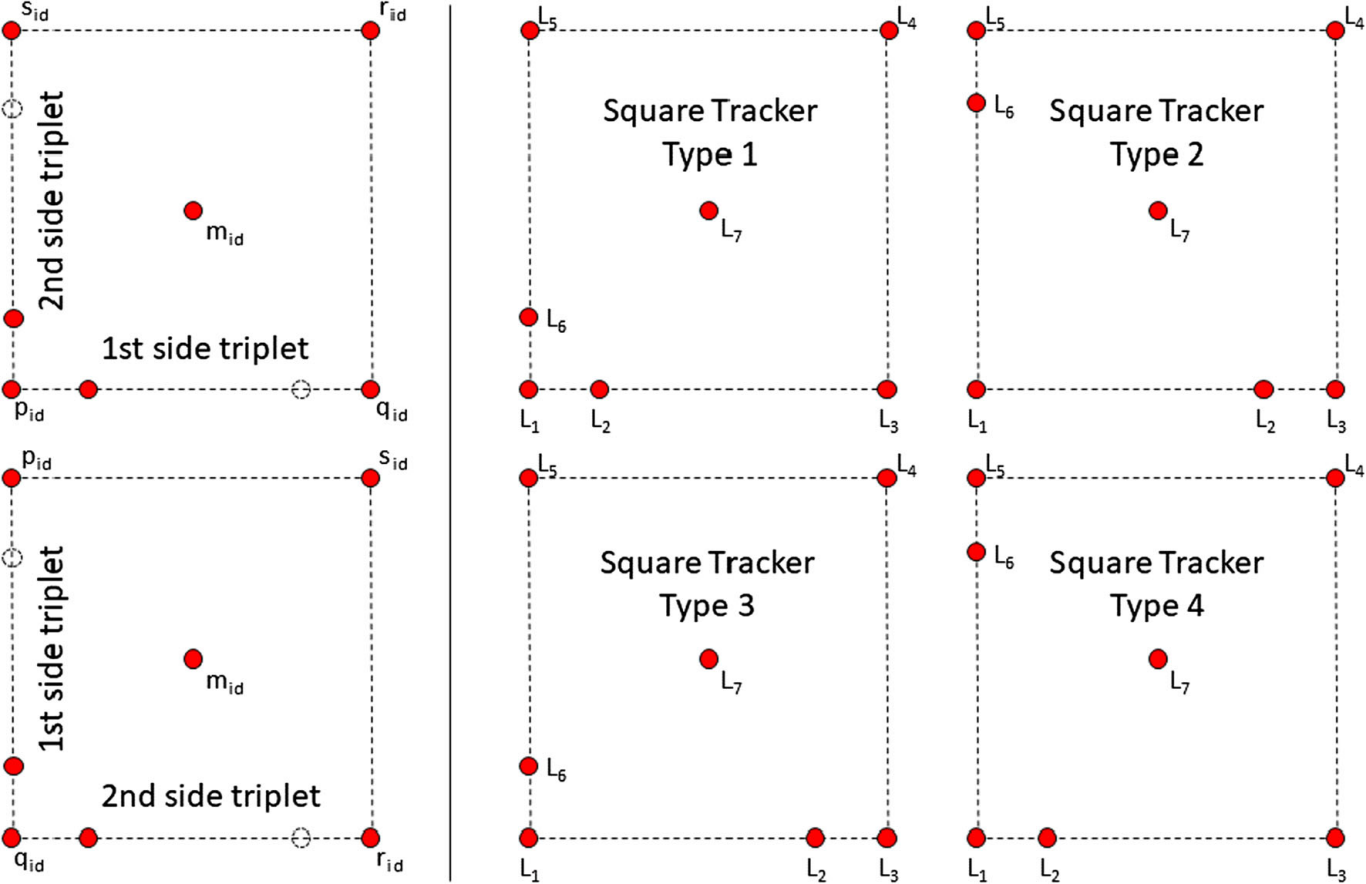
The two different cases for the second side triplet if the first side triplet connects $p_\mathrm{id}$ and $q_\mathrm{id}$ (left) and the four different types of trackers (right)

Figure 4 (left) illustrates the two described cases in counterclockwise order. Other cases are handled analogously.

The handedness of the found constellation is investigated by calculating the determinant of the viewing directions of the first, fifth, and third entry of the current square candidate. If this determinant is negative, the square IDs are stored clockwise and have to be resorted as follows: $\hbox {id}_1/\hbox {id}_6/\hbox {id}_5/\hbox {id}_4/\hbox {id}_3/\hbox {id}_2/\hbox {id}_7$

The last step of the algorithm determines the type of the square tracker by investigating which outer markers are tagged by the tagging markers of the two side triplets (see Fig. 4 right):

- Type 1: Both tagging markers tag $L_1$.
- Type 2: Both tagging markers do not tag $L_1$.
- Type 3: Tagging marker $L_2$ tags $L_3$ and tagging marker $L_6$ tags $L_1$.
- Type 4: Tagging marker $L_2$ tags $L_1$ and tagging marker $L_6$ tags $L_5$.

### Pose estimation algorithm

#### Optimization function

**Fig. 5:**
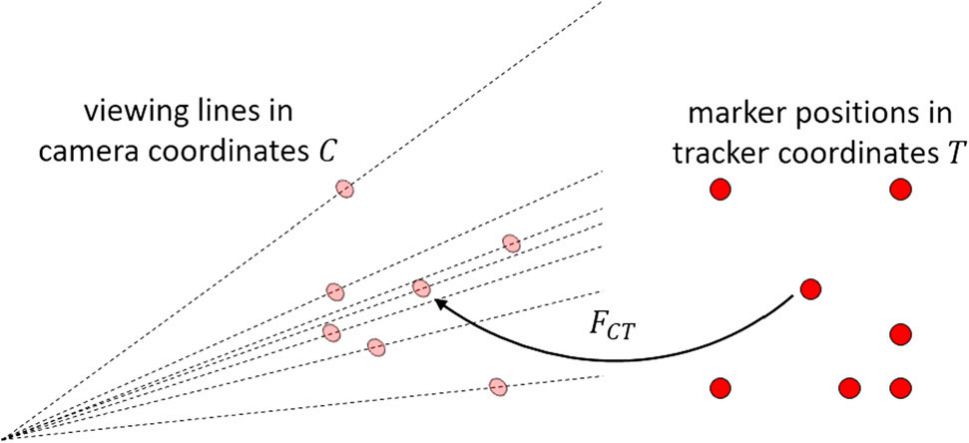
The known marker positions (right) are matched to their corresponding viewing lines (left)

Figure 5 depicts the pose estimation problem: Given a set of straight lines $\mathbf {a}_i + s_i \cdot \mathbf {d}_i$ with normalized direction vectors $\mathbf {d}_i$ in camera coordinates $C$ and a set of corresponding points $\mathbf {p}_i$ with $i = 1 \ldots N$ in tracker coordinates $T$, we search for the rotation matrix $R$ and the translational vector $\mathbf {T}$ such that the sum of squared distances of the transformed points $\mathbf {y}_i = R \cdot \mathbf {p}_i + \mathbf {T}$ to the straight lines gets minimal. With $\mathbf {h}_i = \mathbf {y}_i - \mathbf {a}_i$, the distance of $\mathbf {y}_i$ to $\mathbf {a}_i + s_i \cdot \mathbf {d}_i$ is $\left| \mathbf {h}_i - \left( \mathbf {d}_i^\mathrm{T} \cdot \mathbf {h}_i\right) \cdot \mathbf {d}_i \right|$ and can be written as $\left| \left( E - \mathbf {d}_i \cdot \mathbf {d}_i^\mathrm{T}\right) \cdot \mathbf {h}_i \right|$ where $E$ is the 3$\times$3 identity matrix. Therefore, we have to minimize

1 $$\begin{aligned} f(R, \mathbf {T}) = \sum _{i=1}^{N} \left| \left( E - \mathbf {d}_i \cdot \mathbf {d}_i^\mathrm{T} \right) \cdot \left( R \cdot \mathbf {p}_i + \mathbf {T} - \mathbf {a}_i \right) \right| ^2 \end{aligned}$$

$\mathbf {T}$ can be eliminated by solving $\frac{\partial f}{\partial \mathbf {T}} = 0$ for $\mathbf {T}$ and inserting it back into (1). After parameterizing $R$ with the elements of the corresponding unit quaternion $$\mathbf {q}^\mathrm{T} = \begin{pmatrix} q_0&q_1&q_2&q_3 \end{pmatrix}$$, the optimization function only depends on $\mathbf {q}$ and has the form

2 $$\begin{aligned} f(\mathbf {q}) = \sum _{i=1}^{N} \sum _{j=1}^{3} \left( \mathbf {q}^\mathrm{T} \cdot B_{ij} \cdot \mathbf {q} + k_{ij} \right) ^2 \end{aligned}$$

where $B_{ij}$ are symmetric 4$\times$4 matrices and $k_{ij}$ are scalars depending on $\mathbf {p}_i$, $\mathbf {a}_i$, and $\mathbf {d}_i$. A comprehensive derivation of (2) can be found in [15].

**Fig. 6:**
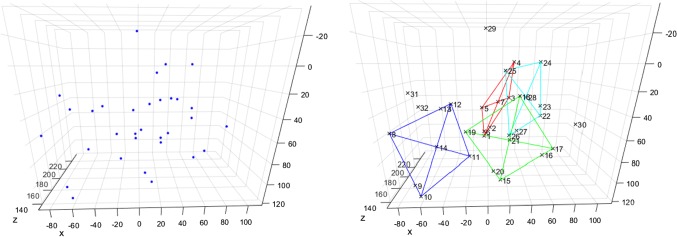
Typical image of four trackers (28 LEDs) together with four extra LEDs seen by the virtual camera (left); the same scene after analyzing the possible tracker constellations (right)

#### Optimization

While Olsson et al. [13] use a branch and bound algorithm for minimizing (2)—unfortunately without presenting its calculation time—the algorithm proposed here uses *Lagrange–Newton Iteration* which turned out to converge extremely fast after a mean of ten iterations. Minimizing $f(\mathbf {q})$ under the constraint $\mathbf {q}^\mathrm{T} \cdot \mathbf {q} = 1$ equals minimizing

3 $$\begin{aligned} F(\mathbf {q}, \lambda ) = \sum _{i=1}^{N} \sum _{j=1}^{3} \left( \mathbf {q}^\mathrm{T} \cdot B_{ij} \cdot \mathbf {q} + k_{ij} \right) ^2 + \lambda \cdot \left( \mathbf {q}^\mathrm{T} \cdot \mathbf {q} - 1 \right) \end{aligned}$$

where the gradient of $F(\mathbf {q}, \lambda )$ has to be zero:

4 $$\begin{aligned}&\mathbf {\nabla }F(\mathbf {q}, \lambda ) = \begin{pmatrix} 2 \lambda \cdot \mathbf {q} + \sum _{i=3}^N \sum _{j=1}^3 4 \cdot v_{ij} \cdot B_{ij} \cdot \mathbf {q} \\ q_0^2 + q_1^2 + q_2^2 + q_3^2 - 1 \end{pmatrix} \nonumber \\&\quad {\mathop {=}\limits ^{!}} \begin{pmatrix} \mathbf {0} \\ 0 \end{pmatrix} \quad \text {with} \quad v_{ij} = \mathbf {q}^\mathrm{T} \cdot B_{ij} \cdot \mathbf {q} + k_{ij} \end{aligned}$$

This nonlinear equation system can be solved iteratively:

5 $$\begin{aligned}&\begin{pmatrix} \mathbf {q}_{\text {new}} \\ \lambda _{\text {new}} \end{pmatrix} = \begin{pmatrix} \mathbf {q}_{\text {old}} \\ \lambda _{\text {old}} \end{pmatrix} - \begin{pmatrix} \varDelta \mathbf {q} \\ \varDelta \lambda \end{pmatrix} \quad \text {with} \quad H_F(\mathbf {q}_{\text {old}}, \lambda _{\text {old}}) \cdot \begin{pmatrix} \varDelta \mathbf {q} \\ \varDelta \lambda \end{pmatrix} \nonumber \\&\quad = \mathbf {\nabla }F(\mathbf {q}_{\text {old}}, \lambda _{\text {old}}) \end{aligned}$$

The elements of the symmetric Hessian matrix $H_F(\mathbf {q}, \lambda )$ are

$$\begin{aligned} H_{kl} = 2 \lambda \cdot \delta _{kl} + 4 \cdot \sum _{i=1}^N \sum _{j=1}^3 \left( 2 \cdot h_{ij}(l) \cdot h_{ij}(k) + v_{ij} \cdot B_{ij}(k,l) \right) \end{aligned}$$

for $0 \le k,l \le 3$ where $\delta _{kl}$ is the Kronecker delta and $\mathbf {h}_{ij} = B_{ij} \cdot \mathbf {q}$. Furthermore, $H_{4k} = H_{k4} = 2q_k$ and $H_{44}$ equals zero.

#### Starting values for the Lagrange–Newton iteration

The above described iterative optimization relies on suitable starting values for $\mathbf {q}$. An inevitable demand for this work was not to rely on guesses or previous calculations. This leads to the question how a sufficient number of uniformly distributed starting quaternions can be arranged on a four-dimensional unit hypersphere such that at least one of them converges to the global minimum.

**Fig. 7:**
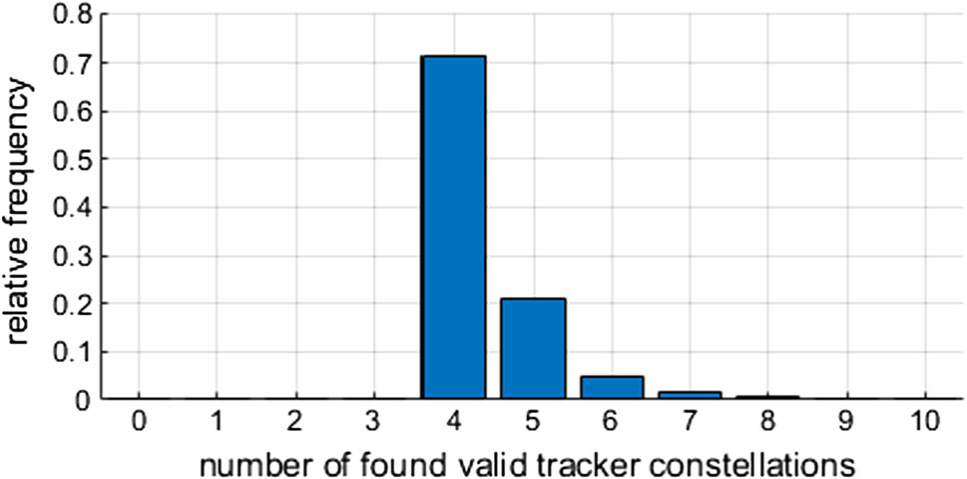
Relative frequency of found valid tracker constellations after 100,000 trials

The solution is using the vertices of the **600-cell or hexacosichora**—one of the six platonic solids in 4D and the equivalent of the icosahedron in 3D. The 120 vertices of the 600-cell are the 16 possible combinations of $(\pm \frac{1}{2}, \pm \frac{1}{2}, \pm \frac{1}{2}, \pm \frac{1}{2})$, the 8 permutations of $(\pm 1, 0, 0, 0)$, and the 96 even permutations of $(\pm \frac{\tau }{2}, \pm \frac{1}{2}, \pm \frac{1}{2\tau }, 0)$ with ($\tau = (1+\sqrt{5})/2$). Since $\mathbf {q}$ and $-\mathbf {q}$ define the same rotation, this results in 60 uniformly distributed quaternions.

**Fig. 8:**
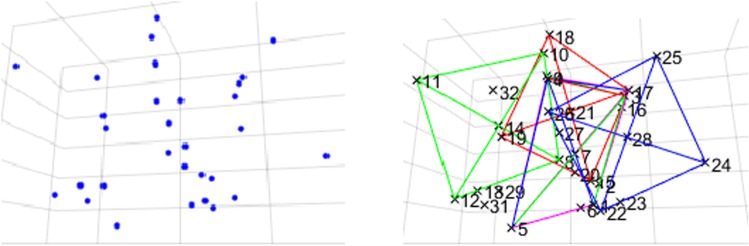
Strongly overlapping (and therefore nearly impossible) constellation with 8 valid tracker constellations

While the derivation of the optimization function (2) is state of the art, uniformly distributing starting rotations as described above has, to the best of the authors’ knowledge, not been presented in the literature before. All experiments have shown that the global minimum is always found if these 60 quaternions are used as starting values.

## Results

In a MATLAB simulation, all four possible tracker types (28 LEDs) together with 4 extra LEDs (possible reflections or other accidental light sources) were randomly placed in front of a virtual camera 100,000 times and analyzed as described in “Marker assignment algorithm” section. Figure 6 (left) shows a typical camera image of the 32 LEDs, and Fig. 6 (right) shows the same scene after analyzing the possible tracker constellations. The simulation was performed without preventing the trackers from overlapping and with the following parameters: 64 mm tracker side length; 150–200 mm distance range of tracker center to projection center; 140 mm maximum distance between tracker center and camera’s principal axis; 85${^{\circ }}$ maximum angle between tracker normal and principal axis.

In 100% of the trials, all four trackers were found. With a relative frequency of 71.6%, only the four trackers and no other valid candidates were found. Furthermore, with a relative frequency of 21.2%, the actual four trackers and one more possible candidate were found. Further details can be found in Fig. 7.

Samples with 7 or more possible candidates only occur if the four randomly placed trackers strongly overlap or if one or more of the four extra LEDs are very close to one of the tracker LEDs, which is rather unlikely in real scenes (see Fig. 8).

**Note 1:** The fact that the algorithm finds more possible candidates than present in the image does not mean that the correct poses of the trackers can not be found. The marker assignment algorithm has to heavily reduce the number of possible candidates before the pose estimation algorithm optimizes the objective function (2) of “Pose estimation algorithm” section. The correct poses correspond to the smallest residual objective function values for the particular tracker types.

**Note 2:** State-of-the-art surgical navigation systems (e.g., NDI Polaris, Stryker FP 6000) triangulate marker positions and calculate transformations by means of point-to-point matching for which at least three markers are needed. If one of these three markers is occluded, the calculation fails. While the minimum number or markers for monocular tracking resp. point-to-line matching is four, the presented algorithms rely on the visibility of seven markers in order to achieve a robust and fast marker assignment which is essential for monocular tracking. The transformation can only be calculated if all seven LEDs are visible.

**Fig. 9:**
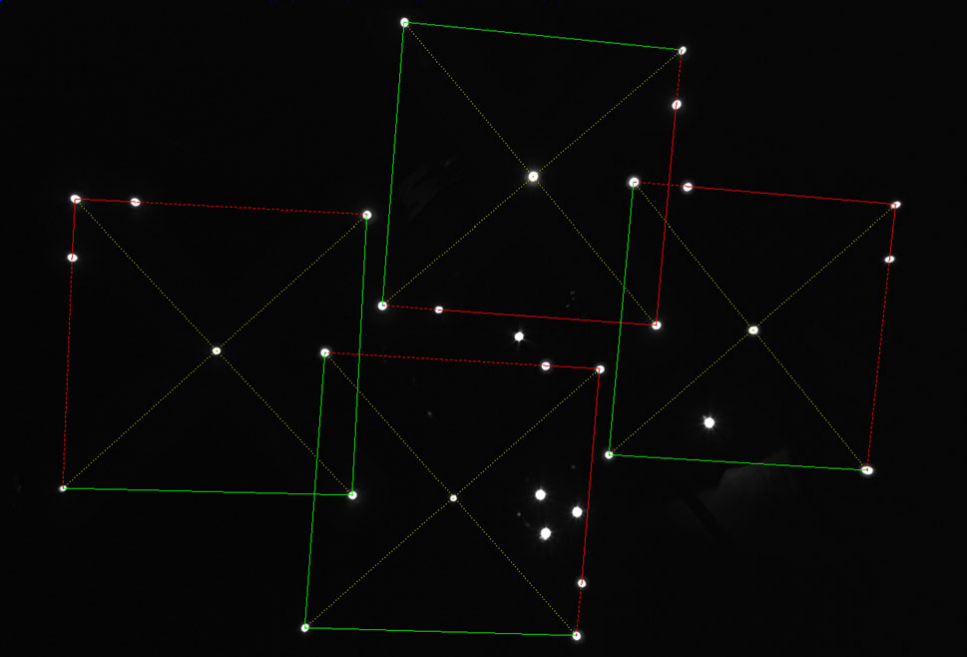
Marker assignment result for four overlapping trackers with five extra light sources

**Table 1:** Calculation time of marker assignment for multiple trackers under real conditions

# of trackers	No accidental light sources (ms)	Seven accidental light sources (ms)
1	6.0	7.0
2	6.5	7.1
3	6.8	7.2
4	7.0	7.5

**Table 2:** Mean deviations of LEDs and tool center points for stereo and monocular tracking

	Stereo	Monocular camera 1		Monocular camera 2	
	LED (mm)	LED (mm)	TCP (mm)	LED (mm)	TCP (mm)
Frontal view	0.10	0.77	0.93	0.48	0.70
45$^{\circ }$ view	0.12	0.61	0.67	0.61	0.64
Random angle	0.11	0.55	0.60	0.46	0.54

**Fig. 10:**
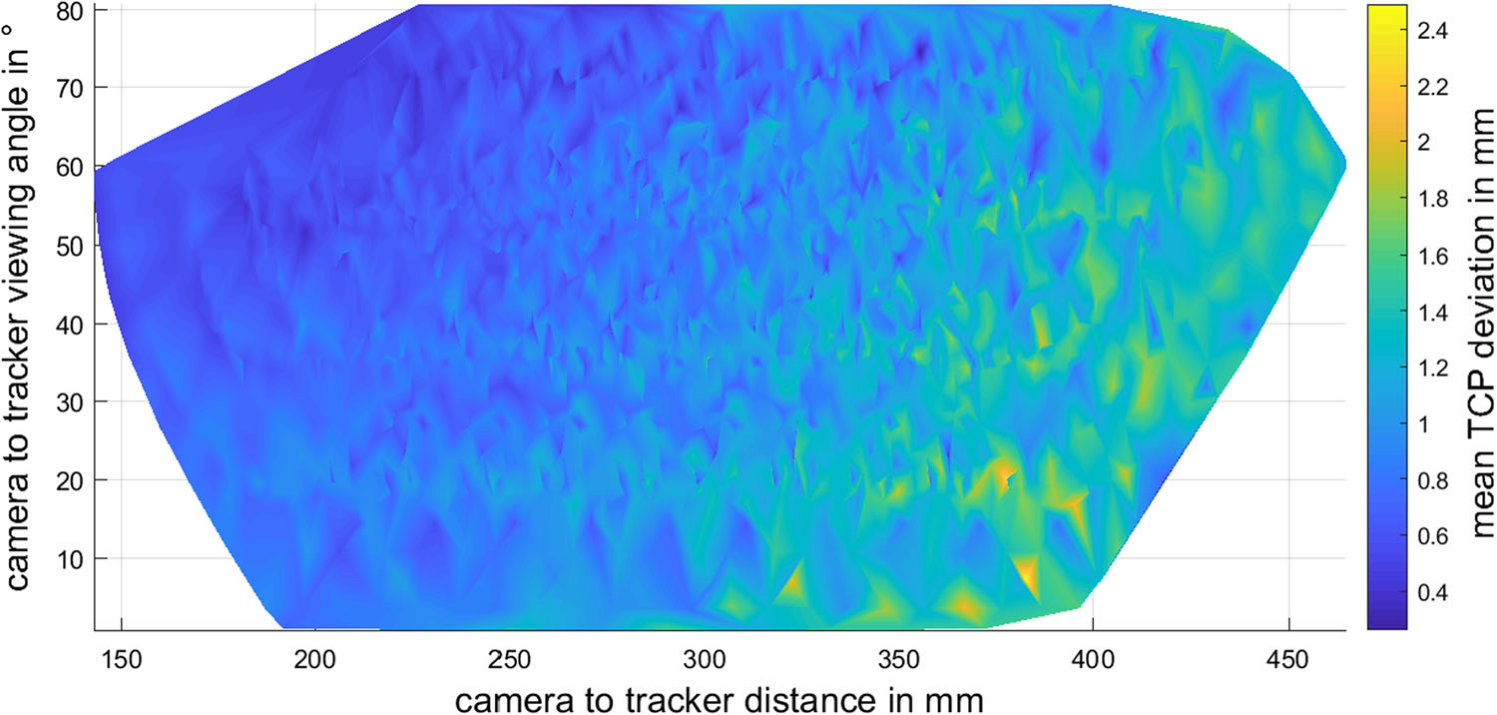
Mean TCP deviations for different distances and viewing angles

All subsequent tests were performed under real conditions. The first test scenario included a calibrated Ximea MQ013MG-E2 with all four tracker types simultaneously placed at distances ranging from 200 to 350 mm. A typical result after marker assignment can be found in Fig. 9. The underlying software was written in C++ using the “Armadillo library for linear algebra & scientific computing” and executed on an Intel i7-6700 at 3.4 GHz. The mean calculation times for marker assignments with and without accidental light sources were measured and are displayed in table 1. The calculation time is small and does not increase significantly with the number of trackers. Although four overlapping trackers are tracked simultaneously together with accidental light sources, the algorithm still robustly finds all trackers.

The mean calculation time for one pose estimation with 60 uniformly distributed start rotations was 5.5 ms per tracker. Therefore, the overall calculation time is below 18 ms for a common use case of two trackers tracked by another peer (6.5 ms for markers assignment, two times 5.5 ms for pose estimation).

### Accuracy tests

In order to compare the proposed peer-to-peer tracking concept in terms of accuracy, three different tests were conducted. They each utilize the same two cameras (The Imaging Source DMM 37UX273-ML), the same camera calibrations, as well as a the same tool center point (TCP) distance of 150 mm.

#### Stereo tracking versus monocular tracking

A stereo camera system is used to calculate the pose of a peer-to-peer tracker utilizing both cameras simultaneously (triangulation and point-to-point matching) and each camera separately (point-to-line matching). This results in three transformations from tracker to camera coordinates. In the first test, the tracker is oriented frontally, under 45° in the second test, and at arbitrary angles in the third. Table 2 shows the measured mean deviations for 100 test positions each. In case of LED deviations, the triangulated LED positions are taken as ground truth and the deviations of the transformed LED positions are used to calculate the mean deviations. For the tool center points located at 150 mm away from the tracker center, the transformation resulting from triangulation was used to calculate the ground truth positions, which are compared to those resulting from point-to-line matching. The results show that the achieved accuracy is practically independent from the viewing angle.

The same test is performed using a linear guide rail together with two rotary joints which are used to automatically control the distance as well as the pitch and yaw angle of the peer-to-peer tracker with respect to the cameras. Again, the stereo transformation is taken as ground truth and the TCP deviations are calculated for more than 2500 positions. Figure 10 shows these deviations for the resulting distances and viewing angles. Please note that the cameras are only calibrated up to a maximum distance of 350 mm which explains higher deviations beyond this limit.

#### Peer-to-peer tracking versus Stryker FP 6000

In the following two tests, the accuracy of the presented peer-to-peer tracking concept is compared to the accuracy of Stryker’s surgical navigation camera FP 6000.

**Table 3:** Pivot test results of 1000 poses for peer-to-peer tracking and the FP6000 navigation system

	Mean (mm)	Std. (mm)	Max (mm)
Peer-to-peer tracking	0.31	0.36	1.14
Stryker FP 6000	0.28	0.31	1.09

**Fig. 11:**
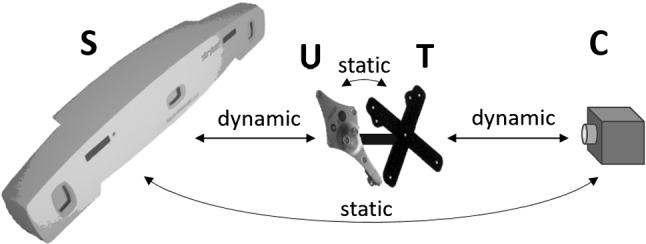
Test setup for the transformation chain calculation

**Fig. 12:**
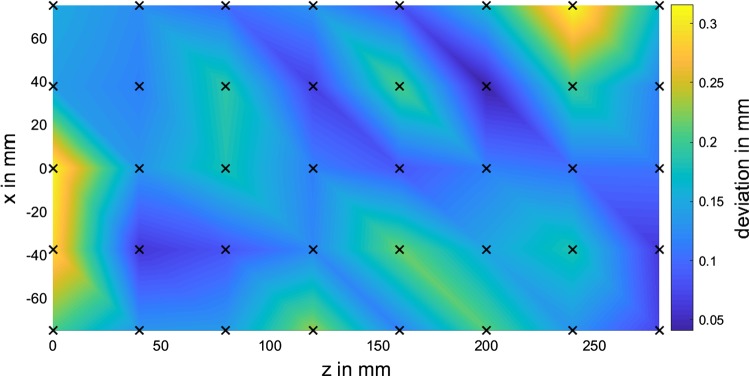
Pivot-to-grid point deviation after point-to-point matching

For the first test, a bearing ball is rigidly attached 150 mm away from the center of a Stryker universal tracker which itself is mounted back to back on a peer-to-peer tracker. Now, the bearing ball is pivoted inside its matching counterpart and for each of the two trackers, 1000 different poses are recorded. Finally, the pivot points are optimized in both coordinate systems and the resulting deviations to the pivot points are calculated for all transformations. Table 3 shows the mean, standard, and maximum deviations for both navigation systems. The results clearly show that the presented peer-to-peer tracking is nearly as accurate as Stryker’s state-of-the-art navigation camera and definitely accurate enough for surgical navigation.

For the second test, Stryker’s universal tracker is again mounted back to back on a peer-to-peer tracker and the two navigation cameras are rigidly attached to the same table such that their relative transformation stays constant (see Fig. 11). Now, 100 arbitrary pairs of corresponding poses $$F_{CT_i}$$ and $$F_{SU_i}$$ are recorded and used to optimize the static transformations $$F_{UT} $$ and $$F_{CS} $$. Afterward, tool center points $$\mathbf {p}_T$$ (still 150 mm away from the tracker center) defined constantly in peer-to-peer tracker coordinates $$T$$ are transformed to navigation camera coordinates $$C$$ directly using $$F_{CT_i}$$ and indirectly using the Stryker transformation as ground truth:

6 $$\begin{aligned} \mathbf {p}_{C_{iP2P}} = F_{CT_i} \cdot \mathbf {p}_T \quad \text {resp.}\quad \mathbf {p}_{C_{i\mathrm{Stryker}}} = F_{CS} \cdot F_{SU_i} \cdot F_{UT} \cdot \mathbf {p}_T \end{aligned}$$

Statistically analyzing the distances $d_i = |\mathbf {p}_{C_{iP2P}} - \mathbf {p}_{C_{i\mathrm{Stryker}}} |$ results in mean deviation of 0.50 mm and a root mean square of 0.57 mm. All in all, it turns out that the accuracy of the presented peer-to-peer tracking concept is definitely comparable to the accuracy of a state-of-the-art surgical navigation system.

#### Peer-to-peer tracking versus ground truth

A thorough comparison of Stryker’s FP 6000 (used in “Peer-to-peer tracking versus Stryker FP 6000” section) against a coordinate measurement machine can be found in [7]. Elfring et al. conclude that the Stryker camera exhibits best-in-class accuracy with a trueness of 0.07 mm.

In order to compare the peer-to-peer tracking concept against a ground truth, two linear guide rails are used to position the pivot mold of the test described in “Peer-to-peer tracking versus Stryker FP 6000” section with an absolute accuracy of 0.02 mm at rasterized grid positions with a grid spacing of 37.5 mm in *x*-direction and 40 mm in *z*-direction. For 40 grid positions, 500 transformations of the pivoted peer-to-peer tracker are recorded. This results in 40 pivot points which are matched to the exact grid positions using point-to-point matching. Figure 12 shows the pivot-to-grid point deviations after matching for all 40 positions. The mean deviation equals 0.14 mm and the maximum deviation is 0.31 mm.

## Conclusion and discussion

In this work and in [15], a new peer-to-peer tracking concept was presented which overcomes the separation between tracking tools and tracked tools. It was shown that one camera per tracker provides sufficient accuracy for surgical navigation. Furthermore, novel algorithms for pose estimation and marker assignment as well as a whole new tracker layout and coding concept based on marker triplets were introduced. Simulations and real experiments showed that the overall concept works fast, accurately, and robustly.

The promising results shown in chapter 3 suggest that the presented approach is a feasible alternative to the current state-of-the-art surgical navigation and has the potential to entail new applications that are not possible using current technology. For example, peer-to-peer tracking opens up the possibility to build tracking chains (one peer tracking the next) and to navigate “around the corner.” Furthermore, the presented trackers only consist of a housing, seven LEDs, and a low-cost camera and can therefore be realized as disposable items which in turn facilitates that they do not have to be sterilizable.

The presented approach also implies advantages with respect to the emerging field of glasses-based augmented reality in the operating theater: Instead of trying to get the tracked see-through glasses (worn by the surgeon) and the tracked tools into the working volume of a conventional surgical navigation system (which is nearly impossible), the glasses and tools only have to be equipped with one of the proposed peer-to-peer trackers in order to realize precise overlays. Corresponding use scenarios can be found in [2, 5]. A similar approach for augmented reality was presented by Vogt et al. [18].

Last but not least, although the concept was developed for surgical navigation, it is also suitable for many other fields where cheap, fast, and accurate methods of tracking are of special interest, e.g., unmanned areal vehicles or self-organizing robot swarms [3, 4]. Future work will concentrate on parallelizing and optimizing the algorithms to ensure an update rate of at least 100 Hz. An achievable goal is reducing the processing time for marker assignment from 7 ms to 5 ms and parallelizing pose estimation such that the poses of all trackers can be calculated in 5 ms.
